# Metabolic effects of elevated temperature on organic acid degradation in ripening *Vitis vinifera* fruit

**DOI:** 10.1093/jxb/eru343

**Published:** 2014-09-01

**Authors:** C. Sweetman, V. O. Sadras, R. D. Hancock, K. L. Soole, C. M. Ford

**Affiliations:** ^1^School of Agriculture, Food & Wine, The University of Adelaide, Australia; ^2^South Australian Research and Development Institute, Australia; ^3^Cell and Molecular Sciences, James Hutton Institute, Invergowrie, Dundee DD2 5DA, UK; ^4^School of Biological Sciences, Flinders University, South Australia

**Keywords:** Enzyme activity, fruit, gene expression, malate, metabolism, ripening, temperature, *Vitis vinifera*.

## Abstract

Berries of the cultivated grapevine *Vitis vinifera* are notably responsive to temperature, which can influence fruit quality and hence the future compatibility of varieties with their current growing regions. Organic acids represent a key component of fruit organoleptic quality and their content is significantly influenced by temperature. The objectives of this study were to (i) manipulate thermal regimes to realistically capture warming-driven reduction of malate content in Shiraz berries, and (ii) investigate the mechanisms behind temperature-sensitive malate loss and the potential downstream effects on berry metabolism. In the field we compared untreated controls at ambient temperature with longer and milder warming (2–4 °C differential for three weeks; Experiment 1) or shorter and more severe warming (4–6 °C differential for 11 days; Experiment 2). We complemented field trials with control (25/15 °C) and elevated (35/20 °C) day/night temperature controlled-environment trials using potted vines (Experiment 3). Elevating maximum temperatures (4–10 °C above controls) during pre-véraison stages led to higher malate content, particularly with warmer nights. Heating at véraison and ripening stages reduced malate content, consistent with effects typically seen in warm vintages. However, when minimum temperatures were also raised by 4–6 °C, malate content was not reduced, suggesting that the regulation of malate metabolism differs during the day and night. Increased NAD-dependent malic enzyme activity and decreased phospho*enol*pyruvate carboxylase and pyruvate kinase activities, as well as the accumulation of various amino acids and γ-aminobutyric acid, suggest enhanced anaplerotic capacity of the TCA cycle and a need for coping with decreased cytosolic pH in heated fruit.

## Summary

Experiments conducted under controlled conditions in vineyards and growth chambers demonstrated day- and night-specific responses of grape berry organic acid levels through altered TCA cycle and amino acid metabolism.

## Introduction

Fruits are specialized sinks that accumulate numerous compounds significant for organoleptic quality such as sugars, organic acids (Kliewer, 1965), pigments (Takos *et al.*, 2006), volatile aromas (Song and Bangerth, 1996; Dunlevy *et al.*, 2010), and flavonoids (Hanlin and Downey, 2009). The occurrence and proportion of such compounds within fruit tissues in the cultivated grapevine *Vitis vinifera* depend on genotype and environment (including management practices), and the interaction between genotype and environment (Kliewer, 1967; Jackson and Lombard, 1993; Downey *et al.*, 2006; Deluc *et al.*, 2007). Although vine and fruit growth and development can be partially controlled through horticultural practices, environmental conditions represent an uncontrollable source of variation in quality that can exhibit effects over subsequent seasons (Sadras and Moran, 2013). Many studies have linked increases in temperature to earlier phenological events in grape berry development, with the potential to greatly affect fruit and wine characteristics (Duchene and Schneider, 2005; Petrie and Sadras, 2008; Ramos *et al.*, 2008; Webb *et al.*, 2011). One of the clearest relationships between temperature and fruit quality occurs with grape berry acidity, whereby high temperatures reduce the concentration of organic acids (Kliewer, 1973).

Developing grapes display distinct patterns of organic acid accumulation and degradation, as reviewed by Ford (2012). Tartrate and malate are predominant at all stages of development and represent the most significant influences on the acidity and pH of the juice (Morris *et al.*, 1983). Grapes accumulate malate until berries undergo a metabolic shift at véraison, making it available as a potential source of carbon for respiration, gluconeogenesis, and other pathways during ripening (Ruffner, 1982). The net loss of malate reduces fruit titratable acidity (Kliewer, 1965) and influences the sugar-acid balance. The loss of malate from grape berries in response to heating has been attributed to increased degradation during ripening rather than decreased synthesis pre-véraison (Ruffner *et al.*, 1976), influencing winemaking processes annually across the globe. However studies are yet to unequivocally determine the biochemical and molecular mechanisms by which increased malate degradation occurs in response to elevated vineyard temperature, and how downstream metabolic pathways are affected. The aims of the present work were therefore to (i) identify temperature elevation strategies that influence fruit organic acid content, and (ii) examine the effects on gene transcripts and activities of key enzymes involved in organic acid metabolism, and to use a metabolomic approach to examine the broader impacts of altered berry malate metabolism.

## Materials and methods

### Experimental design and sample collection

Experiments were conducted with field-grown and potted vines of *V. vinifera* (cv. Shiraz). In the field, untreated controls at ambient temperature were compared with longer and milder warming (2–3 °C differential for three weeks; Experiment 1) or shorter and more severe warming (4–6 °C differential for 11 d; Experiment 2), and in controlled environments potted vines were exposed to control (25/15 °C) and elevated (35/20 °C) temperature conditions for 11 d (Experiment 3).

### Field experiments

Two field trials (Experiments 1 and 2) were conducted during the 2008/09 season using North–South facing, own-rooted vines (Shiraz, clone NSW15) established in 1997 at SARDI’s Nuriootpa Research Station in the Barossa Valley, South Australia (34°S, 134°E, 274m AMSL). Vines were spur-pruned to 40–50 nodes per vine and drip irrigated weekly from mid-December. Phenological development was assessed weekly using the E-L scale of Coombe (1995). Differential temperature regimes were applied at three developmental stages nominally defined as “pre-véraison”, targeting young fruit that were rapidly accumulating malate (E-L 31); “véraison”, initiated just before fruit softening when berries contained peak quantities of malate (E-L 34); and “ripening”, initiated approximately 1–2 weeks after the véraison treatments had ended, at an intermediary level of total soluble solids (TSS) and once a significant portion of malate had been lost from the fruit (E-L 36).

Experiment 1. The longer and milder temperature elevation treatment utilized open-top chambers: polycarbonate panels arranged in a tent-like structure below canopy level across nine vines (Sadras and Soar, 2009). Open-top chambers were used to passively elevate daytime temperature by 2.3–3.8 °C for three weeks; further details of thermal regimes are described by Sadras and Soar (2009). Three replicate treatments were laid out in a randomized block design. Weekly fruit samples (80–90 berries per replicate) were collected across seven vines, from at least five randomly selected bunches per vine.

Experiment 2. The shorter and more severe temperature elevation treatments utilized (i) closed chambers comprising polycarbonate panels that encased three entire vines (Soar *et al.*, 2009), and (ii) fan-forced heaters aimed at individual bunches (an adaptation of Tarara *et al.*, 2000). Combinations of closed chambers and bunch heaters (Supplementary Fig. S1 at JXB online) were used to emulate an 11-d heat event by increasing temperatures 4–6 °C in relation to controls during the day, the night, or both day and night. At each treatment period two closed chambers were assembled: one to simulate control daytime temperature, similar to ambient, and the other to elevate daytime temperature. Within each of the two chambers, four replicates each of control-temperature and elevated-temperature fan heaters were aimed at bunches that had been tagged at 50% cap-fall (E-L 23) for developmental synchronicity (owing to the smaller sample size). This set-up enabled a two-by-two factorial design, with day temperature regulated at the whole-vine level and night temperature regulated at the individual bunch level. The four temperature conditions each contained four individual bunch replicates, labelled “control”, “heated days”, “heated nights” and “heated days and nights”. “External control” samples were also collected from nearby, untreated vines to measure effects of the experimental apparatus; however, “control” samples were used for statistical analyses of heat effects. Samples of 10 berries were collected from each replicate at the end of each treatment, two weeks after each treatment, at véraison (E-L 35) and at harvest ripeness (E-L 38).

### Controlled environment experiment

Experiment 3. Trials were carried out at The Plant Accelerator, The University of Adelaide Waite Campus, South Australia, using four- and five-year-old potted Shiraz (BVRC12) during the 2011/12 and 2012/13 seasons (different vines for each season). Vines were maintained in a shade-house and transferred to two “Conviron” growth chambers after fruitset (E-L 29). Plants were watered daily (800ml) and a slow-release fertiliser was applied before fruit set. Four control plants and four heated plants were selected based on the presence of at least two bunches of similar size and development. Vines were subjected to an acclimation stage for one week under control conditions (16.5h day length, PAR 1100 µmol m^–2^ s^–1^, 25 °C/15 °C day/night temperature and 40%/80% day/night humidity, with each parameter altered gradually to simulate realistic changes during the day and night in the field). After the acclimation period, heated vines were exposed to 11 d of 35 °C/20 °C day/night temperature whilst other conditions remained unchanged. Treatments were applied at two stages, “pre-véraison” (E-L 31) and “véraison” (E-L 34, once berries began to soften). Samples (8 berries) from each vine were collected at the hottest part of the day, on the first, third, and final days of the elevated temperature treatment, the third day of recovery, and at ripeness when berries began to shrivel.

### Sample collection

Berries were selected based on distribution within each bunch (1:2:1 from apical:median:basal and 1:1:1:1 from anterior:posterior:left:right positions) and removed by cutting through the petiole at the junction between stem and berry. Samples for RNA and enzyme extractions were immediately snap-frozen in liquid nitrogen. Samples for organic acid measurements were taken back to the laboratory before freezing. All frozen berries were ground to a fine powder in a liquid nitrogen-cooled A11 basic mill (IKA, Germany) and stored at –80 °C. Additional berries were collected for determination of TSS using a digital pocket refractometer (Atago, Tokyo).

### Malate quantification

For samples collected in 2008/09, organic acids were extracted from 500mg frozen berry powder according to the method of Melino *et al.* (2009b), diluted (1/10) in 0.1M MOPS (pH 8.0) and used for malate quantification according to Möllering (1974). NADH formation was measured at 340nm in 200 µl assays containing 0.1M MOPS (pH 8.0), 10mM NAD^+^, 50mM glutamate, 3 units of alanine aminotransferase, 2 units of malate dehydrogenase, and 20 µl extract. For samples collected from 2011 onwards, organic acids were extracted and analysed using the method of Sweetman *et al.* (2012).

### Enzyme assays

Active grape berry enzymes were extracted using methods adapted from previous studies (Ruffner and Kliewer, 1975; Walker *et al.*, 1999). Twelve volumes of extraction buffer (0.5M Tris-Cl, pH 8.5 with 200M KCl, 20mM MgCl_2_, 10mM EDTA, 8% (w/v) PEG-4000, 8mM cysteine-HCl, 7mM diethyldithiocarbamate, 5mM DTT, 2% (w/v) PVPP, 0.25% (w/v) BSA, 0.5mM PMSF, and 0.5mM p-aminobenzamidine) were added to 1g of frozen grape berry powder and mixed gently at 4 °C for 15min. After centrifugation (2750 *g*, 5min) to remove cell debris, PEG-4000 was added to a final concentration of 65% (w/v), mixed gently at room temperature until dissolved and centrifuged (30 000 *g*, 15min). Precipitated protein was resuspended to 2ml final volume (5mM Tris-Cl, pH 7.0, with 20mM MgCl_2_, 10mM EDTA, 5mM DTT, 3% (v/v) Triton X-100, 0.5mM PMSF, and 0.5mM ρ-aminobenzamidine), re-centrifuged (3000 *g*, 1min) and the supernatant used in enzyme activity assays. All assays were carried out at 25 °C using a FLUOstar UV/vis plate reader (BMG Labtech, Victoria, Australia), in a final volume of 200 µl and initiated by the addition of the reagent listed last.

NAD-dependent MDH activity was quantified as the rate of NADH oxidation at pH 6.0 (50mM MES), in the presence of 5mM oxaloacetate, as described previously (Ruffner *et al.*, 1976). NADP-dependent MDH activity was quantified as the rate of NADPH oxidation at pH 8.0 (50mM TES), in the presence of 5mM DTT and 5mM oxaloacetate, modified from a previous method (Jacquot *et al.*, 1981). NAD-dependent ME activity was quantified as the rate of NAD reduction at pH 7.4 (50mM TES) in the presence of 5mM MnCl_2_, 5mM DTT, 2.5mM potassium cyanide, 0.3 µM octyl gallate (OG), 5mM malate, and 75 µM coenzyme A (CoA), modified from a previous method (Hatch *et al.*, 1982). CoA is required for activation of mitochondrial NAD-ME activity and a temporary rate in the absence of CoA was ascribed to NAD-MDH activity. NADP-dependent ME activity was quantified as the rate of NADP reduction at pH 6.0 (50mM MES) in the presence of 8mM MnCl_2_, 2.5mM potassium cyanide, 0.3 µM octyl gallate, and 5mM malate, a modification from Ruffner *et al.* (1976). PEPC activity was quantified as the rate of NADH oxidation at pH 8.0 (50mM TES), in the presence of 10mM MgCl_2_, 5mM DTT, 5mM KHCO_3_, 6U MDH (Sigma), and 2.5mM PEP, a modification from Ruffner *et al.* (1976). PEPCK activity was quantified as the rate of NADH oxidation at pH 6.7 (50mM MES), in the presence of 0.1M KCl, 6mM MnCl_2_, 25mM DTT, 90mM KHCO_3_, 6U MDH (Sigma), 6mM PEP, and 1mM ADP, as described previously (Walker *et al.*, 1999). PK activity was quantified as the rate of NADH oxidation at pH 7.2 (50mM TES) in the presence of 70mM KCl, 30mM MgCl_2_, 6U lactate dehydrogenase, 5mM PEP, and 8mM ADP, as described previously (Turner and Plaxton, 2000).

### Quantitative real-time PCR

Grape berry RNA was extracted from 1g frozen berry powder according to Davies and Robinson (1996) and purified according to Melino *et al.* (2009a). Genomic DNA contamination was removed using an on-column DNase digestion with RNase-free DNase I (Qiagen, Australia). RNA quality was assessed by agarose gel electrophoresis and quantified with a Nanodrop spectrometer (Thermo Scientific, Biolab, Australia). First-strand cDNA synthesis was achieved with Superscript III reverse transcriptase (Invitrogen) and oligo(dT)_20_. The resultant cDNA was diluted in DNase-free water and 50ng used for each qRT-PCR assay.

Reactions were prepared in Faststart Universal Probe Master (Rox) master mix (Roche, Australia) with gene-specific primers and Universal ProbeLibrary probes (Roche, Australia) in a final volume of 16 µl (Supplementary Table S1 at JXB online). Thermal cycling conditions for all qRT-PCR involved an initial 95 °C melt step (10min), followed by 45 cycles of: 95 °C (15 s) and 57 °C (1min). Assays were conducted with a C1000 Thermal Cycler fitted with a CFX96 Real-time PCR detection system (BioRad), and analysed using the CFX Manager software (BioRad). Data were normalized to a reference number derived from ubiquitin and ankyrin transcript levels in each cDNA sample.

### Metabolite profiling

Samples (25mg of freeze-dried berry powder) were extracted, derivatized, and quantified by GC/MS as previously described (Foito *et al.*, 2009).

### Statistical analyses

Data were analysed using GraphPad Prism v6.04 (California, USA). For Experiment 1, independent two-tailed t-tests were used. For Experiment 2, two-way ANOVAs with Tukey tests for multiple comparisons and two-tailed t-tests were used for comparisons between treatments and controls, and statistical significance of heat treatments was based on comparisons with the control treatments (not external controls). For Experiment 3, samples from two seasons were analysed for interaction between treatment and season using two-way ANOVAs, then pooled and subjected to t-tests. Non-linear regression was used to compare malate content curves for control and treated berries against TSS. Findings were considered significant when *P*≤0.05.

## Results

### Heating methods

Multiple, complementary strategies were applied both in the field and in controlled environments to manipulate thermal regimes and capture realistic warming-driven reduction of malate content in berries. Passive open-top systems used in this study (Experiment 1) were designed to minimize secondary effects typical of enclosures, allowing for a moderate increase in day temperature that can be applied for extended periods (Sadras and Soar, 2009). Closed chambers were used to attain more severe and better controlled day-time warming, but the artefacts of the enclosure constrained its application to shorter periods (Soar *et al.*, 2009). Closed chambers were used in conjunction with fan heaters (Tarara *et al.*, 2000) that regulated bunch temperature at night (Experiment 2). In addition, growth chambers were used with potted vines (Experiment 3) to complement the warming studies in the field. Table 1 summarizes thermal regimes of Experiment 2 and 3; details for Experiment 1 are given by Sadras and Soar (2009). Temperatures measured during natural heatwave events in Nuriootpa can be seen in Supplementary Fig. S2 at JXB online.

**Table 1.** Temperature conditions in field (Experiment 2) and growth chamber (Experiment 3) treatments Recorded at canopy level for Experiment 2, and measured as ambient temperature within chambers for Experiment 3. Diurnal ranges were calculated as the difference between mean maximum and mean minimum temperatures for each experiment.

Treatment	Mean max. (°C)	Mean min. (°C)	Diurnal range (°C)
Experiment 2, pre-véraison Stage			
External control	19.1	9.1	10.0
Chamber control	26.8	10.1	16.7
Heated day	31.6	9.9	21.7
Heated night	27.6	16.5	11.1
Heated day and night	33.0	16.2	16.8
Experiment 2, véraison Stage			
External control	35.2	12.1	23.1
Chamber control	33.6	12.9	20.7
Heated day	37.0	13.1	23.9
Heated night	34.6	19.6	15.0
Heated day and night	38.1	19.5	18.6
Experiment 3, all Stages			
Control	25.0	15.0	10.0
Heated day and night	35.0	20.0	15.0

As a check for the realism of the experimental set ups, berries were assessed for patterns of fresh weight and sugar accumulation during development and were typical of viticulturally relevant conditions (Supplementary Fig. S3 at JXB online). Heated chambers of Experiment 3 generally exhibited lower humidity and CO_2_ concentration than control chambers during the daylight hours (Supplementary Fig. S4 at JXB online) and the fresh weights of the heated fruit were slightly smaller than those in the control chambers (Supplementary Fig. S3F), suggesting water-deficit as a secondary effect of heating, although plant water status was not measured.

### Malate content

Malate was quantified in berry samples from all three experiments to fulfil the first aim: identifying heating strategies that reduced berry malate content. Malate concentration was determined in whole berries using HPLC or enzyme-linked spectrophotometry and data are presented as the total amount of malate per berry, or malate “content”.

### Field experiments

**Fig. 1.**
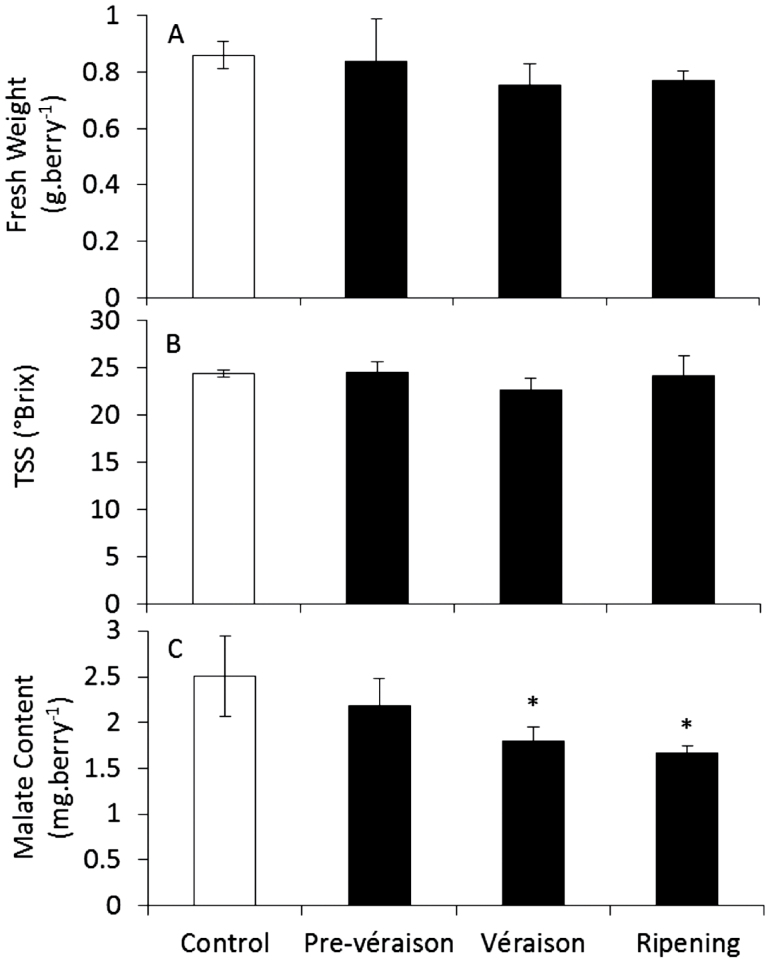
Berry malate content following three-week elevated temperature treatments applied at pre-véraison, véraison, and ripening stages (Experiment 1). Effect on (A) fresh weight, (B) TSS, and (C) malate content of berries collected at harvest (*n*=3±SD). *Significantly different from control (independent t-test, *P*<0.05).

**Fig. 2.**
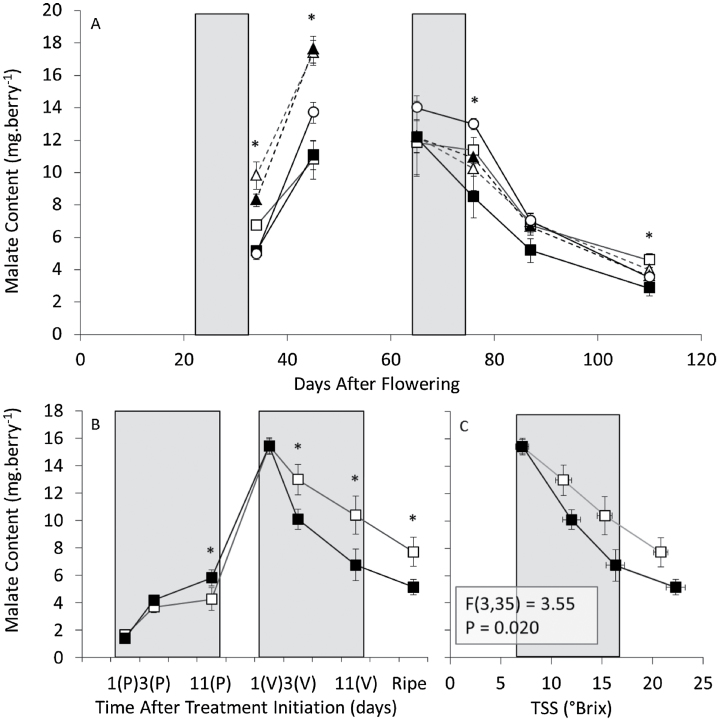
Berry malate content following eleven-day elevated temperature treatments applied at pre-véraison and véraison stages (Experiments 2 and 3). (A) Malate content of berries from Experiment 2 external controls (open circles), control treatments (open squares), heated days (closed squares), heated nights (open triangles), and heated days and nights (closed triangles) for pre-véraison and véraison treatments. (B and C) Malate content from berries of Experiment 3 for control (open squares) and heated (closed squares) vines, plotted against (B) chronological time (days after the initiation of pre-véraison [P] and véraison [V] treatments) and (C) TSS (°Brix) for the véraison treatment. Treatment periods are highlighted in grey. [Samples taken from the final four time-points of (B) and (C) were used for the metabolite analysis shown in Fig. 7]. *n*=8±SD. *Significantly different from samples subjected to the control treatment (independent t-test, *P*<0.05). For (C), the overlay of the curves for control and heated berries was determined by nonlinear regression with least squares.

In Experiment 1, neither fresh weight (Fig. 1A) nor TSS (Fig. 1B) was affected by heating. The mean malate content of berries from the pre-véraison treatment was similar to the control, whereas véraison and ripening treatments had lower malate content (Fig. 1C). In Experiment 2, pre-véraison malate content was significantly higher with night heating but unaffected by day heating (Fig. 2A). This effect was removed by the time the fruit reached véraison, probably owing to the marked metabolic changes, including the switch from net malate accumulation to degradation, the accumulation of sugars, and the loss of fruit photosynthetic capacity (Ollat and Gaudillere, 2000). Day heating at véraison decreased berry malate content, with a 21.5 µmol difference between control (85.8 µmol berry^–1^ or 11.4mg berry^–1^) and heated (63.3 µmol berry^–1^ or 8.5mg berry^–1^) fruit, equating to a 1.4 nmol min^–1^ berry^–1^ greater rate of malate loss over the 11-d treatment period, and remained significantly lower at ripeness (Fig. 2A). Combined day and night heating at véraison did not reduce malate content relative to the control treatment, suggesting that malate loss with warmer days was reduced when berries were also exposed to warmer nights.

### Controlled environment experiment

Experiment 3 was conducted over two seasons (2011–2012 and 2012–2013). As both seasons gave similar results and there was no interaction between treatment and season at any time point (*P*>0.142), these data were pooled. Similar to Experiment 2, heating temporarily increased malate accumulation in pre-véraison fruit and accelerated the loss of malate in ripening fruit (Fig. 2B). The accelerated loss of malate during ripening was uncoupled from TSS (Fig. 2C), and therefore not a result of general advancement in fruit development.

**Fig. 3.**
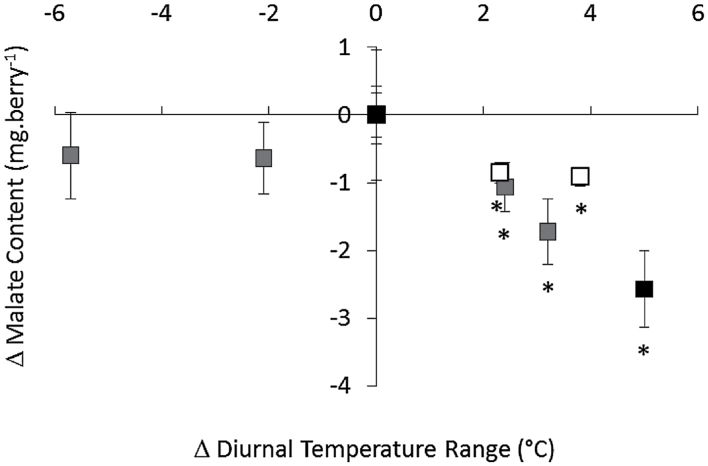
Berry malate content and diurnal temperature range differentials. Differences in diurnal temperature range and malate content between heated and control treatment means for Experiment 1 véraison and ripening treatments (white), Experiment 2 véraison treatment (grey), and Experiment 3 véraison treatment (black). Error bars represent the standard deviation for absolute malate content in the heated samples (or control samples, plotted at 0). Average diurnal temperature range differences for Experiment 1, véraison, and ripening treatments, were estimated from average maximum temperature differentials reported for 2008/09 “véraison” and “pre-harvest” treatments by Sadras and Soar (2009). *n*=3 (Experiment 1), *n*=4 (Experiments 2 and 3). *Significantly different from control (independent t-test, *P*<0.05).

Overall, the loss of malate caused by heating at véraison in Experiment 3 was consistent with losses of malate in field Experiment 1 with heating during véraison and ripening (Fig. 1C) and Experiment 2 with day-only heating at véraison (Fig. 2A). Differences between malate content in heated and control treatments for each experiment were plotted against differences in the diurnal temperature ranges (Fig. 3). Trends suggested that the warming effect on berry malate content may be more significant with increased diurnal temperature range.

As warming in the field and chamber resulted in malate losses that may be typical of hot grape-growing seasons, samples from these experiments were used to explore the second aim: determining the molecular mechanism for accelerated malate loss during ripening and the potential downstream effects on berry metabolism.

### Enzymes of organic acid metabolism

**Fig. 4.**
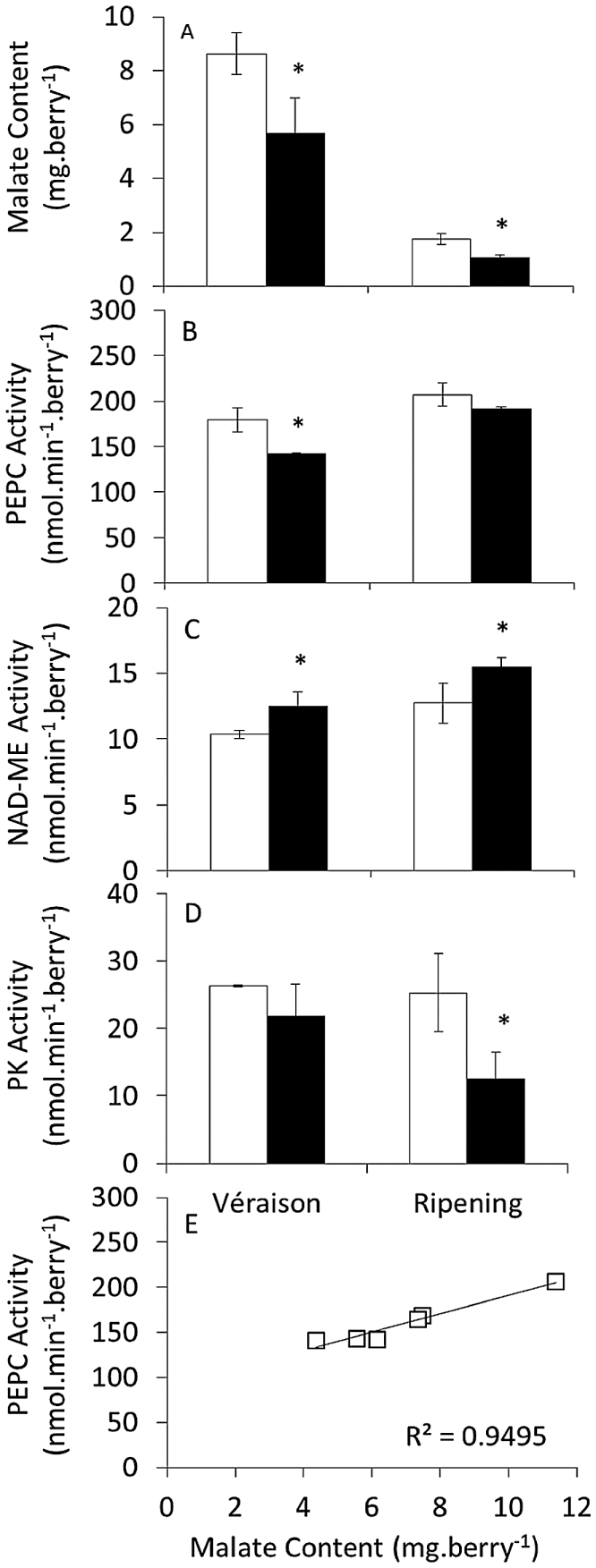
Berry malate content and activities of PEPC, NAD-ME, and PK following three-week elevated temperature treatments applied at véraison and ripening (Experiment 1). (A) Malate content, (B) PEPC activity, (C) NAD-ME activity, and (D) PK activity of berries collected on the final day of the véraison and ripening treatments from control (white) and heated (black) vines. (E) Correlation between malate content and PEPC activity of the véraison treatment. *n*=3±SD. *Significantly different from control (independent t-test, *P*<0.05).

**Fig. 5.**
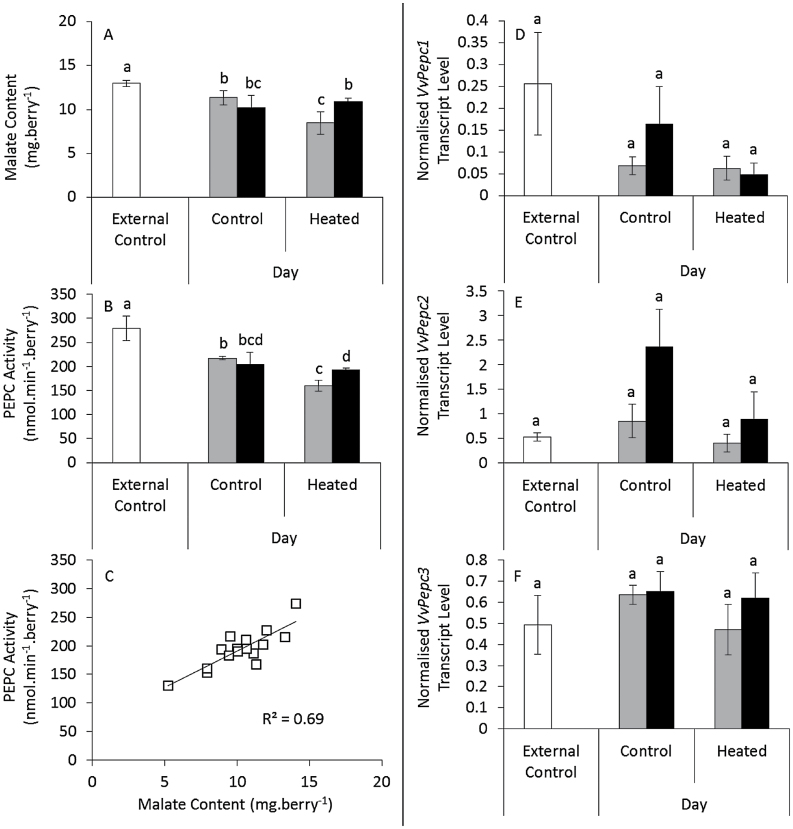
Berry malate content, PEPC activity, and PEPC transcript levels following an eleven-day elevated temperature treatment applied at véraison (Experiment 2). (A) Malate content, (B) PEPC activity, and (C) linear regression between malate content and PEPC activity (left panel) are shown. Transcript levels for (D) *VvPepc1*, (E) *VvPepc2*, and (F) *VvPepc3* are also given (right panel). In column graphs, control and heated day treatments are indicated on the x-axes, with external controls (white), control nights (grey), and heated nights (black) (*n*=4±SD). Columns sharing a lower-case letter are not significantly different (two-way ANOVA with Tukey’s multiple comparisons test; independent t-tests for comparison with external control, *P*<0.05).

**Fig. 6.**
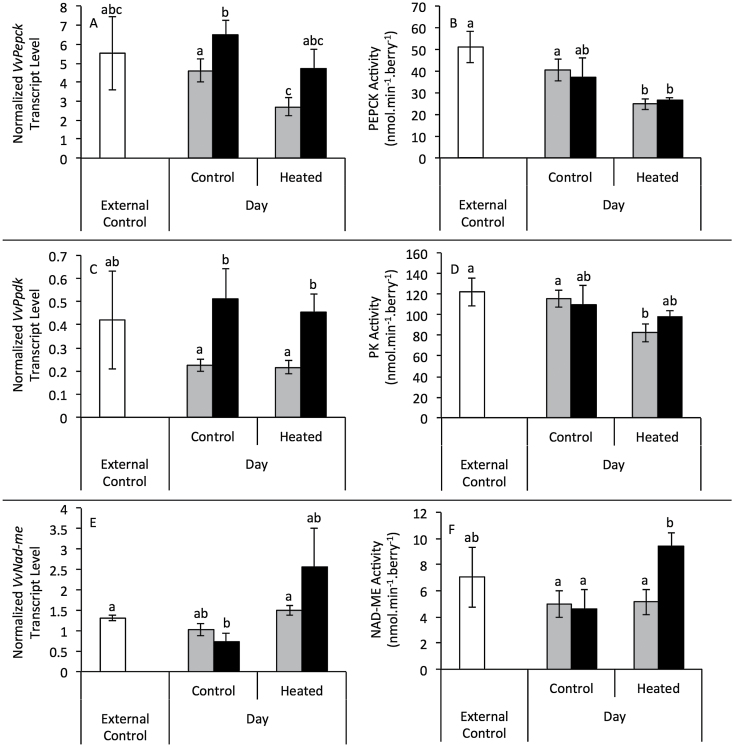
Gene transcript levels and activities of enzymes involved in grape berry malate metabolism following an eleven-day elevated temperature treatment applied at véraison (Experiment 2). (A) *PEPCK* transcript level and (B) PEPCK activity (top panel), (C) *PPDK* transcript level and (D) PK activity (middle panel), and (E) *NAD-ME* transcript level and (F) NAD-ME activity (lower panel) (*n*=4±SD). Control and heated day treatments are indicated on the x-axis, with external controls (white), control nights (grey), and heated nights (black). (n=4±SD). Columns sharing a lower-case letter are not significantly different (two-way ANOVA with Tukey’s multiple comparisons test; independent t-tests for comparison with external control, P<0.05).

Berries exposed to three-week warming at véraison and ripening in the field (Experiment 1) had lower malate content (Fig. 4A), PEPC activity (Fig. 4B), increased NAD-ME activity (Fig. 4C), and decreased PK activity (Fig. 4D) compared with untreated controls. Furthermore, a positive linear correlation was observed between malate content and PEPC activity (Fig. 4E). Berries exposed to 11-d heating at véraison in the field (Experiment 2) had lower PEPC activity with warmer days, unless nights were also warmer, again correlating with malate content (Fig. 5A–C). Slight changes in transcript levels of three PEPC transcripts were not significant (Fig.5D–F). PEPCK transcript (*VvPepck*) and activity decreased in response to day heating (Fig. 6A, B) and although transcript levels of *VvPepck* and a putative pyruvate, orthophosphate dikinase (PPDK) gene (*VvPpdk*) increased with night heating (Fig. 6A, C), this was not reflected in the activity of the PEPCK enzyme (Fig. 6B), whereas PPDK activity could not be detected. PK activity also decreased in response to elevated day temperature (Fig. 6D). Combined day and night heating in Experiment 2 led to increased NAD-ME transcript level and enzyme activity (Fig. 6E, F). NADP-ME, NADP-MDH, and NAD-MDH activities were approximately 60, 70, and 2500 nmol min^–1^ berry^–1^, respectively and unaffected by warming (Supplementary Fig. S5 at JXB online).

### Metabolite pools

**Fig. 7.**
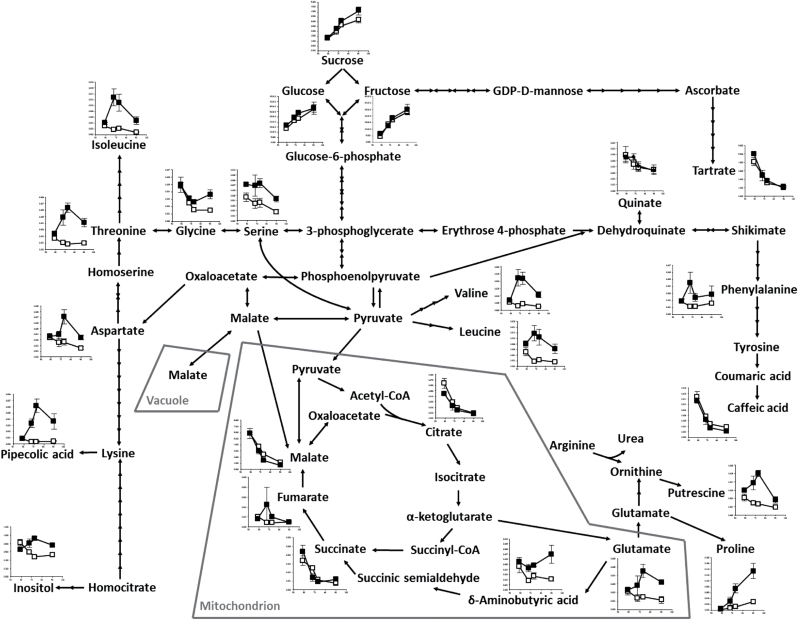
Metabolite pools following an eleven-day elevated-temperature treatment of potted Shiraz vines applied at véraison (Experiment 3). Time-series graphs demonstrate relative concentrations (to internal standard, ribitol) of compounds within control (open squares) and heated (closed squares) berries from (I) the first day of treatment, (II) the third day of treatment, (III) the final day of treatment and (IV) harvest ripeness (i.e. samples taken at the final four timepoints of the véraison treatment as shown in Fig. 2B, C). *n*=4±SD.

To explore potential downstream effects of malate catabolism in heated grapevine berries, metabolite profiling by GC/MS was undertaken on developing fruit (Fig. 7; Supplementary Table S2 at JXB online). Increases in relative concentrations of sucrose and the amino acids valine, leucine, serine, glycine, aspartate, threonine, isoleucine, glutamate, proline, and γ-aminobutyric acid (GABA) were observed. Protein degradation was unlikely to drive the changes in amino acid pools as there was either no difference, or slightly increased protein yield from enzyme extracts of fruit exposed to warming in the field (data not shown), and there were unequal changes between individual amino acids. The non-polar, neutral amino acids proline, valine, leucine, and isoleucine were strongly up-regulated in heated fruit, whereas increases in glutamate, aspartate, serine, and glycine, although significant, were more modest (Fig. 7). Increases in pipecolic acid, putrescine, and inositol, and a small decrease in caffeic acid were also observed.

## Discussion

### Day and night heating, and heating at different developmental stages elicit different responses in malate metabolism

Although season-long warming by approximately 1–2 °C with open-top chambers did not affect Shiraz juice titratable acidity, pH (Sadras *et al.*, 2013), or berry malate content (unpublished data), shorter heat treatments in the current study led to significant changes in malate content, which generally increased with heating during early berry development, and decreased with heating at véraison and ripening stages. Specifically, malate losses were observed with heating at véraison and ripening stages of field Experiment 1 (Fig. 1C), véraison day-only heating in field Experiment 2 (Fig. 2A), and véraison heating in controlled-environment Experiment 3 (Fig. 2B, C). Common to all of these treatments, in addition to the increase in maximum day temperature, was an increase in diurnal temperature range that was comparable to the 3.4 °C increase in average diurnal temperature ranges observed during natural heatwave events in Nuriootpa (Supplementary Fig. S2 at JXB online). In Experiment 2, heating during the night or during both day and night resulted in decreased diurnal temperature ranges and no significant decrease in malate content relative to control berries (Fig. 3). Therefore, malate regulatory mechanisms differ not only with developmental stage, but also between day and night cycles, with increased sensitivity to heating when day temperature is increased to a higher degree than night temperature during ripening. This finding contradicted previous studies where malate losses were observed with heated days regardless of night temperature (Kliewer, 1968, 1971, 1973); however, three major differences separate the previous studies from the present one. Firstly, the present study matched the continuous change between maximum and minimum temperatures in the vineyard (Supplementary Fig. S4 at JXB online), and mimicked heating events that may occur during a typical grape-growing season (Supplementary Fig. S2 at JXB online), whereas previous studies applied alternating eight- to sixteen- hour blocks of day and night temperature, often with diurnal ranges of only 5 °C. Secondly, the present study targeted heating events to specific stages of berry development and ripening as opposed to the entire fruit ripening stage. Thirdly, the bunch-specific night heating strategy used in Experiment 2 differs from the whole-vine heating strategies of previous studies. Varying CO_2_, humidity, and light levels owing to differences in the design of Experiment 1 (Soar *et al.*, 2009), Experiment 2 (Sadras and Soar, 2009), Experiment 3 (Supplementary Fig. S4), and previous experiments (Kliewer, 1968) may account for some variation in malate content between these treatments. The heated growth chambers used in Experiment 3 demonstrated decreased humidity and CO2 levels relative to control growth chambers during the day. Increased levels of berry inositol and pipecolic acid, which are involved in plant defence and stress responses (Loewus and Loewus, 1983; Zeier, 2013), may be due to high temperature or to potential water deficit conditions in the heated growth chamber; therefore, some caution is required when interpreting these results. Nevertheless, water loss is a direct effect of heating in the vineyard, and the effects on malate content in berries of potted vines were consistent with those seen in field experiments.

Inherent differences in activities and gene expression profiles of malate-metabolizing enzymes in grapes (Hawker, 1969; Terrier *et al.*, 2005; Deluc *et al.*, 2007; Pilati *et al.*, 2007; Sweetman *et al.*, 2009) and their differing temperature optima (Lakso and Kliewer, 1975b), are likely to cause differences in the response of malate to heating at different developmental stages and during the day compared with night. Although a previous study with potted Cabernet Sauvignon vines could not attribute decreased titratable acidity in heated fruit to altered activities of malate-metabolizing enzymes (Ruffner *et al.*, 1976), some activities were altered in response to warming of potted and field-grown Shiraz vines in the present study, and observed changes in metabolite pools were also used to explore endpoints of metabolic pathways altered by heating.

### Regulation of malate synthesis with warming

Malate in the berry is synthesised from phospho*enol*pyruvate (PEP) via PEPC and MDH, competing with PK and the ultimate step of glycolysis (Sweetman *et al.*, 2009). Both MDH and PEPC activities are present throughout berry development (Hawker, 1969; Diakou *et al.*, 2000), although PEPC transcript levels are generally favoured during early development (Sweetman *et al.*, 2009). High rates and negligible changes in the activities of NADP-MDH and NAD-MDH with elevated temperature suggested that neither of these enzymes regulate malate content in response to warming. However, the activity of PEPC in fruit of vines exposed to day heating during véraison and ripening correlated positively with malate content (Figs 4E and 5E) and the 50 nmol min^–1^ berry^–1^ decrease in PEPC activity could account for the degree of malate loss observed in fruit from heat-treated vines (approximately 1.4 nmol min^–1^ berry^–1^ based on the difference of 2.9mg berry^–1^ between control and heated fruit over 11 d). In pre-véraison berries, PEPC activity decreased with day heating regardless of night temperature (data not shown), but malate content did not correlate with changes in PEPC activity, confirming that PEPC is not rate-limiting for pre-véraison malate accumulation (Ruffner, 1982). Activity of purified PEPC from immature Carignane grape berries increased with temperatures up to a maximum of 38–40 °C (Lakso and Kliewer, 1975a, 1978). Therefore, night heating in Experiment 2, which resulted in mean minimum temperatures of 19 °C relative to 13 °C in controls, may have considerably increased PEPC activity and hence malate content during the night, whereas day heating, which resulted in mean maximum temperatures of 37 °C relative to 33.6 °C in controls may not significantly affect activity.

The three PEPC genes of grapevine are differentially expressed during development (Sweetman *et al.*, 2009), and at least one of these (*VvPepc2*) showed a similar pattern to PEPC activity and malate content, and furthermore decreased in response to heating, according to data from a previous study (Carbonell-Bejerano *et al.*, 2013). However, this transcript is not regulated diurnally (Carbonell-Bejerano *et al.*, 2014; Rienth *et al.*, 2014). According to the online grapevine co-expression database VtcDB (Wong *et al.*, 2013), putative PEPC genes (VIT_19s0015g00410 and VIT_19s0014g01390) may be co-expressed with PK genes (VIT_13s0074g00210 and VIT_16s0050g02180), and in the present study PK activity was down-regulated with heating in a similar manner to PEPC activity. Co-ordinated regulation of these activities, which both utilize PEP, could markedly affect glycolytic flux in heated berries.

### Regulation of malate degradation with warming

Heating increased the activity and transcript level of NAD-dependent ME (NAD-ME) in pre-véraison (data not shown), véraison, and ripening treatments (Fig. 6E, F). In pre-véraison fruit, up-regulation of NAD-ME activity coincided with increased malate content and therefore the respiration rate of malate may be exceeded by its synthesis and sequestration in the vacuole, whereas in véraison and ripening fruit, up-regulation of NAD-ME coincided with decreased malate content and may play a role in net malate loss. NAD-ME-catalysed conversion of mitochondrial malate to pyruvate provides NADH to the mitochondrial electron transport chain and acetyl-CoA (from pyruvate) to the TCA cycle. Malate can also enter the TCA cycle directly through the activity of mitochondrial MDH, again supplying NADH to the mitochondrial electron transport chain. However, this enzyme cannot be measured from whole berry extracts, owing to the presence of additional isoforms in other compartments (Taureilles-Saurel *et al.*, 1995). Although the mitochondrial malate concentration in grape berries is unknown, transcript levels of several mitochondrial dicarboxylate/tricarboxylate transporters increased with warming (Rienth *et al.*, 2014), suggesting increased import of malate into the mitochondria for respiration. A decrease in CO_2_ solubility at higher temperatures may also promote the decarboxylation of malate by NAD-ME (Lakso and Kliewer, 1978), whereas the increase in fumarate, an NAD-ME activator (Tronconi *et al.*, 2010), and decrease in citrate, an NAD-ME inhibitor (Wedding, 1989), observed in heated berries (Fig. 7), would further increase activity *in vivo*. Increased NAD-ME activity may therefore facilitate increased respiration rates observed in heated grapevine clusters (Palliotti *et al.*, 2005), utilizing malate as a fuel source during ripening. Observed increases in numerous amino acids, typically those derived from pyruvate (valine, leucine, serine, and glycine), oxaloacetate (aspartate, threonine, and isoleucine) and α-ketoglutarate (glutamate, proline, and GABA) indicate a change in TCA cycle regulation that could be a result of the observed increase in NAD-ME activity.

Centeno *et al.* (2011) demonstrated the wide-ranging effects of perturbing mitochondrial malate metabolism using antisense tomato lines. Tomato fruits possessing decreased fumarase and mitochondrial MDH activities contained respectively lower and higher levels of malate, and amino acid levels were generally reduced in both circumstances, probably owing to the interruption of the TCA cycle. Antisense MDH lines showed some evidence of increased flux through NAD-ME and PDH as a result of the increase in malate concentration, enabling a malate-driven supply of acetyl-CoA to rescue the TCA cycle. The redox state of the fruit was also altered, which subsequently affected the regulation of starch metabolism, causing a strong negative correlation between malate and starch concentrations across all lines. Although grape berries are a non-climacteric fruit that contain negligible levels of starch (Downton & Hawker, 1973), the present study demonstrates that increased flux through the TCA cycle and NAD-ME caused by elevated temperatures accelerated the utilization of malate and could increase the anaplerotic capacity of the TCA cycle for amino acid biosynthesis. The down-regulation of PEPCK transcript level and activity measured in this, and other recent studies (Rienth *et al.*, 2014), suggests that the utilization of malate in supplementing the TCA cycle may be favoured over its use in gluconeogenesis. As such, increased PEPCK activity does not contribute to accelerated malate degradation in berries of heated vines and the significant increase in sucrose levels, which occurs in the absence of change in glucose and fructose levels, is therefore a result of increased import into the berry rather than gluconeogenesis. Together, these studies highlight the importance of the regulation of malate and TCA cycle enzymes in amino acid and carbohydrate metabolism in developing fruits, both climacteric and non-climacteric.

Accumulation of the non-proteinogenic amino acid GABA in berries of heat-treated vines (Fig. 7) supports the link between organic acid degradation and amino acid synthesis through TCA cycle intermediates and the GABA shunt as seen in post-harvest citrus fruit and, along with the observed increases in proline and the polyamine putrescine, may be symptomatic of an oxidative stress response (Ye *et al.*, 1997; Bouche and Fromm, 2004; Ozden *et al.*, 2009; Sun *et al.*, 2013). Proline accumulates as a normal function of berry ripening (Stines *et al.*, 1999; Deluc *et al.*, 2007); however warming resulted in hyper-accumulation (Fig. 7), similar to drought- and salinity-stressed grapevine leaves (Cramer *et al.*, 2007). Up-regulation of pyrroline-5-carboxylate synthetase transcript in heated berries (Rienth *et al.*, 2014), could account for the increased proline levels observed in the present study, and increases the supply of NADP^+^ to the cytosol (Kohl *et al.*, 1990), thereby stimulating NADP-ME that could contribute to malate metabolism in the cytosol. Putrescine declined during ripening of control berries, as seen in other fruits (Aizat *et al.*, 2014), whereas up-regulation with heating suggests negative regulation of ethylene biosynthesis, also seen in other fruits (Ketsa *et al.*, 1999). Glutamate, a precursor of putrescine, GABA, proline, and arginine, was higher in berries from heat-treated vines and therefore indicates the metabolic pathways by which these compounds were probably synthesized. Glutamate in heated grapes could be generated from α-ketoglutarate through increased TCA cycle activity. In addition to its role in the GABA shunt, glutamate can also inhibit PEPC expression (Sugiharto and Sugiyama, 1992), and thus may simultaneously play a role in the down-regulation of PEPC transcript level and enzyme activity observed in berries of heated vines.

Despite the presence and notable up-regulation of *VvPpdk* transcript levels in grape berries at véraison (Sweetman *et al.*, 2009), PPDK activity, which catalyses the reversible conversion between pyruvate and PEP, is yet to be successfully measured in fruits. The level of PPDK protein in grape berry and other fruits is also low or undetectable (Famiani *et al.*, 2007), although post-harvest peach fruit stored at 39 °C for three days and nights demonstrated increased PPDK protein and decreased PK protein along with decreased malate concentration (Lara *et al.*, 2009). In the present study, *VvPpdk* transcript was up-regulated with night-heating at both pre-véraison (data not shown) and véraison (Fig. 6C) stages. Further work is required to determine whether PPDK is active in grape berries.

### Malate compartmentalization

The developmental control of grape berry malate degradation may be associated with the rate of its release from the vacuole. Tonoplastic dicarboxylate transporters regulate the rate of malate import into plant vacuoles (Etienne *et al.*, 2013), which decreases in response to low cytosolic pH (Pantoja and Smith, 2002). A recently characterized tonoplastic malate and tartrate inward-rectifying transporter gene in grapevine, *VvAlmt9* (De Angeli *et al.*, 2013), demonstrated increased transcription around véraison (Deluc *et al.*, 2007) and may maintain a malate concentration gradient between the vacuole and cytosol during ripening. A strong positive link between fruit acidity and another tonoplastic transporter gene, *Ma1*, was reported in apple (Bai *et al.*, 2012). Decreased transcript levels (and presumably activities) of *VvAlmt4-like* (a putative grapevine *Ma1* homolog; VIT_01s0011g03290), *VvAlmt9* (VIT_17s0000g03850), and a tonoplast dicarboxylate transporter (VIT_00s0187g00130) in leaves and berries exposed to elevated temperatures, based on publicly available microarray data (Dash *et al.*, 2012; Liu *et al.*, 2012; Rienth *et al.*, 2014), could result in decreased retention of malate in the berry vacuole, thereby increasing the availability of the acid for degradation in other cellular compartments. The ripening-induced up-regulation of vacuolar proton-pumping pyrophosphatases (PPases), which transfer protons from cytosol to vacuole and generate an electrical potential gradient across the tonoplast, may also regulate malate transport into the vacuole (Terrier *et al.*, 2001). Characterisation of the activities of these transporters during pre-véraison, véraison and ripening stages would help to elucidate their roles in malate regulation, and a special effort should be made to measure their response to changes in temperature and cytosolic pH.

Upon the release of malate from the vacuole around véraison, the formation of pyruvate from malate is likely to be favoured over glycolysis, as the latter generates protons (Sakano, 1998), whereas NAD-ME activity, which is activated at low pH, ensures continued TCA cycle activity when MDH is inhibited by low pH and oxaloacetate build-up (Wedding, 1989). PPDK activity will be favoured over PK in the conversion of PEP to pyruvate at low pH, as the former consumes two protons whereas the latter consumes only one (Edwards *et al.*, 1985; Sakano, 1998), and both will be favoured over PEP carboxylation to OAA by PEPC, which uses HCO_3_^–^ from the proton-generating carbonic anhydrase (Sakano, 1998). At véraison, the up-regulation of NAD-ME activity (unpublished data), increase in PPDK transcript level, and decrease in PEPC transcript level (Sweetman *et al.*, 2009; Sweetman *et al.*, 2012) suggests that véraison is accompanied by cytosolic acidification, whereas up-regulation of *VvPpdk* transcript level and NAD-ME activity and down-regulation of PK and PEPC activities in grape berries exposed to elevated temperature in the present study suggest that this mechanism may also be utilized during warming or heat stress. In addition, the heat-induced accumulation of GABA, which is formed through glutamate decarboxylase activity and induced by cytosolic H^+^ and Ca^2+^ (Kinnersley and Turano, 2000), consumes protons in the cytosol and may further enable cellular pH to remain within physiological limits.

## Conclusions

The difference between pre-véraison, véraison, and ripening responses of malate to heating could not be explained by changes in transcript levels or activities of enzymes explored in the present study, and may be due instead to differences in the regulation of malate compartmentalization between these developmental stages, as pre-véraison berries undergo net malate accumulation through vacuolar sequestration whereas ripening berries undergo net malate degradation upon its release from the vacuole. Grape berries that were heated by 4–10 °C for 11 d or 2–4 °C for three weeks during véraison and ripening showed significant losses of malate, although when night temperatures were also raised such that the diurnal temperature range between treatment and control was either maintained or decreased, there was no significant malate loss compared with controls. PEPC activity, which correlated positively with malate content in treatments applied during véraison and ripening, could play a role in the day- and night-specific regulation of malate in response to temperature, and therefore malate synthesis remains important in spite of the net loss of the acid at this time. However, increased amino acid pools and NAD-ME activity implicate an increase in anaplerotic flux through the TCA cycle as the cause of accelerated malate degradation in grapes of heated vines, which may compete with gluconeogenesis at higher temperatures and warrants further investigation using flux analysis. Overall, the decrease in PEPC and PK activities and increase in NAD-ME activity, *VvPpdk* transcript level, and glutamate and GABA levels suggested a malate-driven response to cytosolic acidification in fruit that were heated during véraison and ripening.

Whilst some grapevine cultivars contain higher berry malate content at harvest owing to varying patterns of accumulation and degradation throughout development and ripening (Kliewer *et al.*, 1967; Diakou *et al.*, 1997), it is imperative to determine whether warming will affect the acidity of these cultivars in a similar manner to Shiraz. Further research should target the effects of heating on malate compartmentalization and cytosolic pH homeostasis in pre-véraison, véraison, and ripening fruit, to elucidate the differential regulation of malate metabolism between developmental stages and day/night cycles. The utilization of transgenic plants is necessary for further characterisation of the roles of fruit PEPC, NAD-ME, and TCA cycle enzymes in regulating organic acid levels and primary metabolic pathways, as demonstrated in tomato (Centeno *et al.* 2011), while the *VvPpdk* gene also warrants further examination based on up-regulation of the transcript at véraison and its coordinated response to day/night temperature shifts. Meanwhile, the effect of altering cytosolic pH on the mobilization and catabolism of malate and on the activities of *VvAlmt9* and *VvAlmt4* gene products could be investigated using fruit cell culture systems. The ultimate goal is to identify grapevine cultivars and practices that maintain fruit sugar-acid balance as well as aroma, flavour and texture compounds with implications for wine properties under changing environmental conditions.

## Supplementary data

Supplementary data are available at *JXB* online

Table S1. List of accession numbers, primer sets and probes used for qRT-PCR.

Table S2. Compounds detected using GC/MS in control and heated fruit from Experiment 3.

Figure S1. Details of heating strategies used in Experiment 2.

Figure S2. Maximum, minimum and diurnal temperature ranges in Nurootpa, South Australia during heatwaves and average conditions from 1999 to 2014.

Figure S3. Effects of elevated temperature treatments on berry fresh weight and TSS across all three experimental designs.

Figure S4. Details of growth conditions and heating regimes used in Experiment 3.

Figure S5. Activities of NADP-ME, NADP-MDH and NAD-MDH following eleven-day elevated temperature treatments at véraison for Experiment 2.

Supplementary Data
