# Supplementary material for: Metabolic effects of elevated temperature on organic acid degradation in ripening Vitis vinifera fruit
Source: J Exp Bot. 2014 Sep 1;65(20):5975–88. doi: 10.1093/jxb/eru343 (PMC4203137; doi:10.1093/jxb/eru343)
Supplement: Supplementary Data [file supp_65_20_5975__index.html]

Metabolic effects of elevated temperature on organic acid degradation in ripening Vitis vinifera fruit — Metabolic effects of elevated temperature on organic acid degradation in ripening Vitis vinifera fruit — Supplementary Data 

# Metabolic effects of elevated temperature on organic acid degradation in ripening *Vitis vinifera* fruit

## Supplementary Data

Data files

**Files in this Data Supplement:**

- Supplementary Data - Supplementary Data
