# Supplementary material for: Metabolic effects of elevated temperature on organic acid degradation in ripening Vitis vinifera fruit
Source: J Exp Bot. 2014 Sep 1;65(20):5975–88. doi: 10.1093/jxb/eru343 (PMC4203137; doi:10.1093/jxb/eru343)
Supplement: Supplementary Data [file supp_eru343_jexbot128306_file001.pdf]

Metabolic effects of elevated temperature on organic acid degradation in ripening *Vitis vinifera* fruit

Sweetman, C., Sadras, V.O., Hancock, R.D., Soole, K.L., Ford, C.M.

Supplementary Tables and Figures

Table S1: List of accession numbers, primer sets and probes used for qRT-PCR. Probe numbers are from the Human Universal Probe Library (Roche, Australia).

Gene	Accession #	Protein	Primer Sequences (5'-3')	Probe#
PEPC1	XM_002280533	XP_002280569	F: cgccagttattatcaagtggaa<br>R: caagcacagagtgatctatagcaga	34
PEPC2	XM_002280806	XP_002280842	F: atgcccgacttgctgttagt<br>R: aatagaggaaccacccgtagc	113
PEPC3	XM_002285405	XP_002285441	F: aatagaggaaccacccgtagc<br>R: caagaaggtccttgtgtccag	25
PEPCK	XM_003635567	XP_003635615	F: cattgatctctcaagggagaaag<br>R: tcgaacaccacattttccaac	147
NAD-ME	XM_002266661	XP_002266697	F: gactgactttgtcaaccaaaagatag<br>R: gccatattaagcacaccaatcc	4
PPDK	XM_002278776	XP_002278812	F: tgggaaccatgattgagattc<br>R: gaagaattctgcctccttcg	164
Ankyrin	XM_002283462	XP_002283498	F: ggttatggcaggaaggagtg<br>R: ggtgtcttgccatccatgtt	120
Ubiquitin	XM_002273532	XP_002273568	F: gtggccacagcaaccagt<br>R: gcaacctccaatccagtcat	143

Table S2: Compounds detected using GC/MS (Experiment 3). Values highlighted in bold indicate significant differences between berries of control and heated vines. Values were considered significantly different when the log2 difference was <-0.5 or >0.5, and p ≤0.05 according to 2-tailed t-tests. Also available as an .xls spreadsheet.

	59 D.A.F. (0 days of heating)						63 D.A.F. (3 days of heating)					
Compound	Control	S.D.	Heated	S.D.	log_2 difference	t-test	Control	S.D.	Heated	S.D.	log_2 difference	t-test
**Valine**	0.00551	0.00075	0.00674	0.00037	0.291	0.189	0.00295	0.00065	0.02219	0.00561	**2.910**	**0.014**
Ethanolamine	0.01943	0.00501	0.01218	0.00194	-0.674	0.226	0.00419	0.00040	0.00824	0.00213	0.974	0.111
**Leucine**	0.00525	0.00140	0.00808	0.00083	0.622	0.133	0.00172	0.00018	0.01182	0.00278	2.777	0.011
Glycerol	0.00360	0.00045	0.00473	0.00037	0.391	0.102	0.00336	0.00025	0.00494	0.00038	**0.556**	**0.013**
**Isoleucine**	0.00303	0.00060	0.00422	0.00009	0.478	0.096	0.00185	0.00026	0.01280	0.00285	**2.789**	**0.009**
**Proline**	0.00291	0.00063	0.00512	0.00036	**0.814**	**0.023**	0.00731	0.00255	0.03091	0.01331	2.081	0.199
**Glycine**	0.00499	0.00105	0.00488	0.00040	-0.031	0.928	0.00247	0.00031	0.00312	0.00025	0.334	0.155
Succinic acid	0.00167	0.00027	0.00220	0.00036	0.400	0.283	0.00124	0.00012	0.00071	0.00021	-0.799	0.073
Dihydroxypropanoic acid	0.00866	0.00161	0.01015	0.00049	0.229	0.409	0.00557	0.00083	0.00658	0.00051	0.241	0.341
**Fumaric acid**	0.00052	0.00004	0.00040	0.00005	-0.369	0.115	0.00022	0.00005	0.00113	0.00086	2.371	0.332
**Serine**	0.00463	0.00081	0.00716	0.00030	**0.631**	**0.026**	0.00339	0.00091	0.00692	0.00207	1.028	0.170
**Piperidinecarboxylic acid**	0.00083	0.00022	0.00087	0.00018	0.070	0.889	0.00035	0.00005	0.00332	0.00067	**3.256**	**0.004**
**Threonine**	0.00172	0.00038	0.00244	0.00039	0.508	0.228	0.00103	0.00028	0.00490	0.00119	**2.247**	**0.020**
**β-alanine**	0.00063	0.00009	0.00072	0.00014	0.198	0.597	0.00071	0.00019	0.00172	0.00044	1.287	0.078
**Malic acid**	5.84767	0.83655	5.90177	0.18806	0.013	0.952	3.76015	0.33866	3.03517	0.53760	-0.309	0.297
**Aspartic acid**	0.00342	0.00042	0.00367	0.00024	0.104	0.616	0.00257	0.00073	0.00396	0.00061	0.624	0.195
**g-aminobutyric acid**	0.00453	0.00124	0.00548	0.00087	0.273	0.555	0.00181	0.00026	0.00427	0.00074	**1.237**	**0.020**
Threonic acid	0.02314	0.00451	0.02850	0.00151	0.300	0.303	0.01164	0.00205	0.01273	0.00342	0.129	0.794
**Glutamic acid**	0.00217	0.00043	0.00232	0.00023	0.094	0.775	0.00136	0.00039	0.00282	0.00115	1.052	0.274
Tartaric acid	4.10258	0.47001	5.05466	0.22746	0.301	0.118	2.49530	0.16884	2.44492	0.64016	-0.029	0.942
Phenylalanine	0.00184	0.00026	0.00189	0.00035	0.043	0.902	0.00114	0.00022	0.00453	0.00149	1.991	0.066
Trihydroxypentanoic acid	0.00127	0.00020	0.00127	0.00022	-0.009	0.980	0.00077	0.00016	0.00154	0.00070	1.008	0.320
**Gluconic acid 1**	0.01058	0.00149	0.01871	0.00183	**0.822**	**0.014**	0.00599	0.00066	0.00577	0.00121	-0.055	0.877
Gluconic acid 2	0.00548	0.00063	0.00680	0.00050	0.310	0.152	0.00502	0.00083	0.02416	0.01888	2.267	0.350
**Putrescine**	0.00210	0.00041	0.00299	0.00011	0.511	0.083	0.00143	0.00015	0.00386	0.00082	**1.428**	**0.027**
Citric acid	0.06354	0.00934	0.04442	0.00328	-0.516	0.102	0.02957	0.00413	0.02230	0.00773	-0.407	0.439
Quinic acid	0.00299	0.00068	0.00279	0.00033	-0.096	0.807	0.00219	0.00038	0.00278	0.00026	0.345	0.246
Fructose 1	2.57005	0.80991	3.70153	0.36335	0.526	0.250	7.79427	0.70335	6.82930	0.97273	-0.191	0.452
Fructose 2	1.97170	0.60596	2.74923	0.24954	0.480	0.280	6.04415	0.60194	5.78966	0.57065	-0.062	0.769
**Glucose 1**	11.45127	1.50854	13.82281	0.61419	0.272	0.196	16.76545	0.99582	19.44693	2.88395	0.214	0.413
Glucose 2	2.06636	0.37026	2.75219	0.28548	0.413	0.193	4.52420	0.49536	4.45738	0.50934	-0.021	0.928
**Glucaric / Galactaric acid**	0.00049	0.00015	0.00037	0.00009	-0.401	0.523	0.00043	0.00010	0.00063	0.00018	0.558	0.361
**Inositol**	0.82730	0.07558	0.66077	0.03430	-0.324	0.092	0.58946	0.02323	0.80570	0.10235	0.451	0.085
Caffeic acid	0.01485	0.00184	0.01339	0.00091	-0.149	0.505	0.00763	0.00079	0.00640	0.00171	-0.254	0.538
**Glucose-6-phosphate**	0.00073	0.00018	0.00037	0.00005	-0.965	0.108	0.00020	0.00006	0.00045	0.00004	**1.174**	**0.030**
Sucrose	1.28610	0.09631	1.32898	0.10027	0.047	0.768	1.95568	0.24915	2.24188	0.20962	0.197	0.413
n-tetradecanoic acid	0.03754	0.00568	0.02597	0.00203	-0.532	0.103	0.02243	0.00126	0.02375	0.00092	0.083	0.430
**hexadecanoic acid**	0.09941	0.01235	0.05476	0.00353	**-0.860**	**0.013**	0.05836	0.00795	0.04778	0.00565	-0.289	0.320
n-hexadecanoic acid	16.35029	1.97548	10.93913	1.03610	-0.580	0.051	9.43441	0.57552	9.29740	0.72299	-0.021	0.887
n-heptadecanoic acid	0.06430	0.00778	0.04260	0.00674	-0.594	0.080	0.03542	0.00271	0.03419	0.00464	-0.051	0.828
linoleic acid	33.61466	3.28800	23.77132	3.19611	-0.500	0.075	22.26612	1.56988	19.95808	1.93361	-0.158	0.390
alinoleic acid	1.03090	0.19746	0.75106	0.05149	-0.457	0.219	0.55087	0.09373	0.39540	0.00867	-0.478	0.150
**octadecanoic acid**	23.30121	2.78715	14.19274	1.83895	**-0.715**	**0.034**	15.39399	0.43354	12.71401	0.99666	-0.276	0.049
**2OH-hexadecanoic acid**	0.18634	0.01759	0.19179	0.00803	0.042	0.788	0.12189	0.00987	0.10434	0.00651	-0.224	0.188
n-octadecanoic acid	6.61192	0.97354	4.30639	0.55186	-0.619	0.085	3.74094	0.39347	3.15451	0.24119	-0.246	0.251
n-eicosanoic acid	0.22731	0.02472	0.21122	0.02245	-0.106	0.647	0.17196	0.01027	0.13530	0.00577	-0.346	0.021
n-heneicosanoic acid	0.03558	0.00396	0.02895	0.00680	-0.298	0.431	0.02254	0.00294	0.01915	0.00153	-0.235	0.347
eicosanol	0.04336	0.01117	0.03477	0.01021	-0.319	0.591	0.04306	0.00690	0.08453	0.02312	0.973	0.136
n-docosanoic acid	0.16384	0.01212	0.17424	0.00590	0.089	0.469	0.14142	0.01194	0.13494	0.00880	-0.068	0.678
n-docosanol	0.08783	0.00711	0.09327	0.00649	0.087	0.593	0.06031	0.01443	0.05402	0.00314	-0.159	0.685
n-tricosanoic acid	0.04192	0.00701	0.04059	0.00110	-0.047	0.857	0.02829	0.00179	0.02563	0.00110	-0.142	0.252
n-tricosanol	0.00969	0.00195	0.00819	0.00198	-0.242	0.609	0.00507	0.00118	0.00751	0.00145	0.566	0.240
n-tetracosanoic acid	0.19263	0.02011	0.27185	0.01908	0.497	0.029	0.12942	0.00978	0.14968	0.01841	0.210	0.369
**n-tetracosanol**	0.33944	0.04345	0.49182	0.02576	**0.535**	**0.023**	0.20225	0.02693	0.23778	0.04640	0.233	0.532
n-pentacosanoic acid	0.02546	0.00527	0.01996	0.00195	-0.351	0.366	0.01321	0.00270	0.01275	0.00173	-0.051	0.891
2OH-tetracosanoic acid	0.15853	0.01420	0.16146	0.00696	0.026	0.859	0.11769	0.00925	0.09760	0.00356	-0.270	0.089
n-hexacosanoic acid	0.09250	0.01156	0.10885	0.00233	0.235	0.215	0.06453	0.00597	0.06260	0.00938	-0.044	0.868
n-hexacosanol	1.07719	0.16248	1.26564	0.08770	0.233	0.347	0.60338	0.08956	0.57796	0.10965	-0.062	0.863
**n-heptacosanol**	0.03667	0.00333	0.02940	0.00154	-0.319	0.095	0.01889	0.00401	0.01844	0.00285	-0.035	0.929
**n-octacosanoic acid**	0.06996	0.00990	0.06852	0.00565	-0.030	0.904	0.05046	0.00383	0.05442	0.00971	0.109	0.718
n-octacosanol	0.70044	0.10497	0.70537	0.05277	0.010	0.968	0.37731	0.04463	0.37776	0.06622	0.002	0.996
stigmasterol	0.05408	0.00454	0.04966	0.00644	-0.123	0.595	0.03745	0.00233	0.03186	0.00402	-0.233	0.275
b-sitosterol	3.19522	0.34859	2.71080	0.09947	-0.237	0.230	1.94587	0.13348	1.75894	0.10433	-0.146	0.312
**n-triacontanoic acid**	0.03763	0.00574	0.02259	0.00219	**-0.736**	**0.050**	0.02598	0.00165	0.02276	0.00407	-0.191	0.491

	71 D.A.F. (11 days of heating)						90 D.A.F. (ripe fruit)					
Compound	Control	S.D.	Heated	S.D.	log_2 difference	t-test	Control	S.D.	Heated	S.D.	log_2 difference	t-test
**Valine**	0.00423	0.00086	0.02190	0.00421	**2.373**	**0.006**	0.00271	0.00036	0.01063	0.00195	**1.972**	**0.007**
Ethanolamine	0.00298	0.00016	0.00354	0.00028	0.249	0.133	0.00439	0.00197	0.00377	0.00023	-0.221	0.764
**Leucine**	0.00226	0.00044	0.01046	0.00283	**2.213**	**0.029**	0.00167	0.00031	0.00634	0.00172	**1.929**	**0.036**
Glycerol	0.00403	0.00017	0.00384	0.00048	-0.071	0.718	0.00421	0.00064	0.00361	0.00035	-0.222	0.441
**Isoleucine**	0.00219	0.00045	0.01100	0.00296	**2.327**	**0.026**	0.00085	0.00019	0.00485	0.00120	**2.517**	**0.017**
**Proline**	0.01172	0.00578	0.07460	0.01559	**2.670**	**0.009**	0.02869	0.00806	0.13391	0.02626	**2.222**	**0.009**
**Glycine**	0.00155	0.00027	0.00266	0.00029	**0.782**	**0.031**	0.00149	0.00019	0.00366	0.00060	**1.297**	**0.014**
Succinic acid	0.00057	0.00003	0.00044	0.00009	-0.364	0.216	0.00039	0.00013	0.00061	0.00007	0.654	0.189
Dihydroxypropanoic acid	0.00271	0.00039	0.00341	0.00040	0.331	0.259	0.00129	0.00022	0.00124	0.00035	-0.055	0.912
**Fumaric acid**	0.00021	0.00002	0.00050	0.00008	**1.280**	**0.012**	0.00025	0.00006	0.00023	0.00003	-0.115	0.792
**Serine**	0.00355	0.00087	0.00739	0.00088	**1.056**	**0.021**	0.00173	0.00048	0.00429	0.00057	**1.310**	**0.014**
**Piperidinecarboxylic acid**	0.00039	0.00011	0.00617	0.00112	**3.994**	**0.002**	0.00041	0.00008	0.00364	0.00125	**3.154**	**0.041**
**Threonine**	0.00083	0.00020	0.00637	0.00072	**2.947**	**0.000**	0.00100	0.00022	0.00413	0.00066	**2.041**	**0.004**
**β-alanine**	0.00075	0.00015	0.00177	0.00026	**1.246**	**0.015**	0.00064	0.00015	0.00151	0.00031	**1.233**	**0.047**
**Malic acid**	2.44038	0.13028	1.45138	0.10834	**-0.750**	**0.001**	1.17503	0.05712	0.69178	0.06300	**-0.764**	**0.001**
**Aspartic acid**	0.00265	0.00054	0.00713	0.00129	**1.427**	**0.018**	0.00155	0.00023	0.00343	0.00043	**1.142**	**0.008**
**g-aminobutyric acid**	0.00268	0.00061	0.00481	0.00024	**0.844**	**0.018**	0.00207	0.00037	0.00696	0.00174	**1.751**	**0.033**
Threonic acid	0.00733	0.00154	0.00569	0.00074	-0.365	0.375	0.00323	0.00030	0.00410	0.00097	0.343	0.426
**Glutamic acid**	0.00149	0.00029	0.00455	0.00076	**1.604**	**0.009**	0.00114	0.00039	0.00323	0.00029	**1.506**	**0.005**
Tartaric acid	1.56869	0.20779	1.83431	0.19157	0.226	0.384	1.16937	0.11965	1.00939	0.11200	-0.212	0.367
Phenylalanine	0.00112	0.00026	0.00244	0.00059	1.119	0.087	0.00157	0.00033	0.00285	0.00123	0.858	0.354
Trihydroxypentanoic acid	0.00079	0.00010	0.00069	0.00012	-0.204	0.518	0.00050	0.00008	0.00063	0.00021	0.334	0.580
**Gluconic acid 1**	0.00364	0.00020	0.00505	0.00053	0.471	0.047	0.00479	0.00104	0.00446	0.00071	-0.102	0.803
Gluconic acid 2	0.00486	0.00060	0.00490	0.00010	0.013	0.947	0.00584	0.00155	0.00969	0.00450	0.730	0.450
**Putrescine**	0.00135	0.00021	0.00500	0.00037	**1.894**	**0.000**	0.00093	0.00006	0.00182	0.00041	0.969	0.073
Citric acid	0.01827	0.00267	0.01457	0.00098	-0.327	0.240	0.00913	0.00090	0.00807	0.00094	-0.177	0.447
Quinic acid	0.00193	0.00033	0.00207	0.00035	0.096	0.791	0.00174	0.00040	0.00170	0.00026	-0.037	0.929
Fructose 1	9.32155	0.41205	10.25022	0.29627	0.137	0.117	12.62596	1.20666	13.80294	1.88101	0.129	0.617
Fructose 2	7.67278	0.34046	8.44702	0.42094	0.139	0.203	10.68163	1.27530	11.43884	2.06685	0.099	0.766
**Glucose 1**	18.08993	0.73109	22.60245	0.72810	**0.321**	**0.005**	26.11457	3.56714	26.33348	5.30222	0.012	0.974
Glucose 2	5.29248	0.48003	6.46737	0.33694	0.289	0.092	7.07678	0.38812	7.29243	0.72293	0.043	0.801
**Glucaric / Galactaric acid**	0.00053	0.00008	0.00107	0.00014	**1.022**	**0.015**	0.00110	0.00030	0.00241	0.00062	1.137	0.105
**Inositol**	0.48223	0.03715	0.92289	0.05394	**0.936**	**0.001**	0.52556	0.02239	0.76314	0.02987	**0.538**	**0.001**
Caffeic acid	0.00488	0.00056	0.00343	0.00056	-0.509	0.119	0.00371	0.00023	0.00226	0.00051	**-0.715**	**0.041**
**Glucose-6-phosphate**	0.00028	0.00010	0.00022	0.00011	-0.346	0.706	0.00023	0.00002	0.00027	0.00009	0.204	0.727
Sucrose	2.60145	0.03337	3.07095	0.27788	0.239	0.144	3.17671	0.27685	4.11315	0.41381	0.373	0.109
n-tetradecanoic acid	0.01810	0.00390	0.02674	0.00171	0.563	0.089	0.01672	0.00276	0.01390	0.00191	-0.266	0.433
**hexadecanoic acid**	0.05904	0.00573	0.04334	0.00359	-0.446	0.059	0.03446	0.00859	0.02759	0.00330	-0.321	0.483
n-hexadecanoic acid	8.51768	0.84986	7.12189	0.55664	-0.258	0.219	5.11273	0.68709	4.54244	0.25646	-0.171	0.466
n-heptadecanoic acid	0.02709	0.00507	0.01960	0.00242	-0.467	0.231	0.01720	0.00240	0.01774	0.00205	0.045	0.870
linoleic acid	20.83871	3.50570	13.25113	1.83029	-0.653	0.103	9.82869	2.00031	11.40627	2.13798	0.215	0.609
alinoleic acid	0.35857	0.03711	0.33952	0.02706	-0.079	0.693	0.30263	0.02439	0.28763	0.02132	-0.073	0.660
**octadecanoic acid**	12.93133	1.49186	10.33485	0.97201	-0.323	0.195	7.70098	1.25132	7.53204	0.58871	-0.032	0.907
**2OH-hexadecanoic acid**	0.08416	0.01117	0.07931	0.00455	-0.086	0.702	0.06307	0.00586	0.04297	0.00516	**-0.553**	**0.042**
n-octadecanoic acid	3.37816	0.63089	2.33533	0.40169	-0.533	0.213	1.52252	0.27991	1.69057	0.21197	0.151	0.649
n-eicosanoic acid	0.13177	0.01395	0.11362	0.00597	-0.214	0.277	0.10721	0.01619	0.08840	0.00819	-0.278	0.340
n-heneicosanoic acid	0.02193	0.00363	0.02003	0.00407	-0.131	0.739	0.01463	0.00037	0.01439	0.00212	-0.025	0.912
eicosanol	0.03999	0.00995	0.04940	0.00751	0.305	0.479	0.05388	0.01098	0.06255	0.01638	0.215	0.676
n-docosanoic acid	0.12971	0.01329	0.12795	0.00924	-0.020	0.917	0.11731	0.00538	0.10805	0.01494	-0.119	0.581
n-docosanol	0.04581	0.01412	0.04401	0.01279	-0.058	0.928	0.03925	0.00508	0.02425	0.00526	-0.695	0.086
n-tricosanoic acid	0.01795	0.00371	0.01858	0.00079	0.050	0.872	0.01334	0.00086	0.01185	0.00031	-0.171	0.152
n-tricosanol	0.00472	0.00142	0.00347	0.00087	-0.444	0.481	0.00492	0.00088	0.00394	0.00087	-0.321	0.459
n-tetracosanoic acid	0.09726	0.01066	0.10631	0.00232	0.128	0.439	0.06997	0.00330	0.08201	0.01018	0.229	0.303
**n-tetracosanol**	0.12708	0.01792	0.15293	0.01657	0.267	0.330	0.10664	0.01259	0.10799	0.01408	0.018	0.945
n-pentacosanoic acid	0.01001	0.00150	0.00763	0.00120	-0.392	0.260	0.00746	0.00149	0.00532	0.00048	-0.489	0.220
2OH-tetracosanoic acid	0.07650	0.01030	0.09154	0.00620	0.259	0.258	0.06209	0.00514	0.04513	0.00474	-0.460	0.051
n-hexacosanoic acid	0.04489	0.00418	0.05613	0.00178	0.322	0.048	0.04468	0.00206	0.05081	0.00687	0.186	0.425
n-hexacosanol	0.37896	0.04659	0.45314	0.02768	0.258	0.220	0.39493	0.03927	0.34560	0.03600	-0.192	0.390
**n-heptacosanol**	0.01063	0.00127	0.02050	0.00256	**0.948**	**0.014**	0.01747	0.00257	0.01441	0.00276	-0.278	0.448
**n-octacosanoic acid**	0.03952	0.00431	0.06329	0.00301	**0.680**	**0.004**	0.04212	0.00225	0.05820	0.00619	0.467	0.050
n-octacosanol	0.25414	0.02374	0.34324	0.02063	0.434	0.030	0.26418	0.02724	0.25637	0.03655	-0.043	0.870
stigmasterol	0.02070	0.00231	0.01713	0.00136	-0.273	0.231	0.01464	0.00227	0.01293	0.00132	-0.180	0.538
b-sitosterol	1.34919	0.13944	1.35237	0.03188	0.003	0.983	1.09224	0.03048	0.87780	0.04778	-0.315	0.009
**n-triacontanoic acid**	0.01705	0.00338	0.03374	0.00364	**0.985**	**0.015**	0.01363	0.00243	0.02671	0.00182	**0.971**	**0.005**

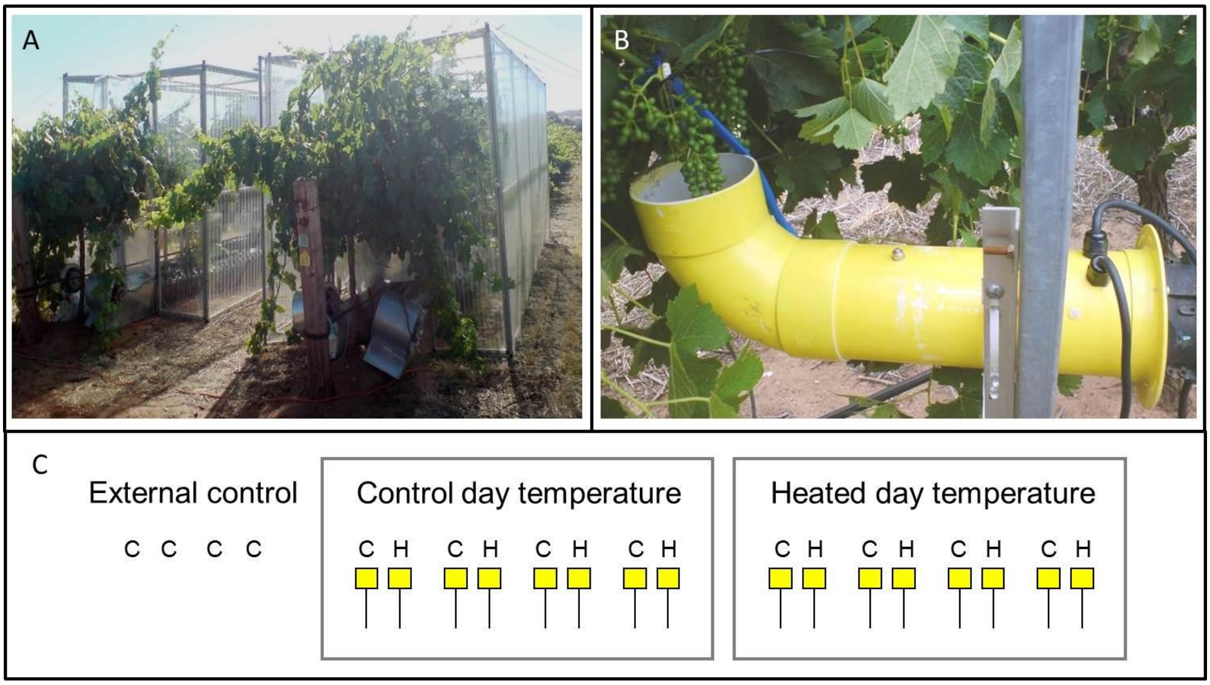

Figure S1: Experiment 2 equipment and design. (A) Closed-top chambers (Soar *et al*., 2009) were used to modulate day temperature around whole vines and (B) fan heaters were used to modulate night temperature around individual bunches. (C) The two-factorial design comprising (i) two chambers, one set to simulate ambient day temperature (Control) and the other to elevate day temperature (Heated), and (ii) eight sets of fan heaters (yellow boxes) per chamber, four set to maintain ambient night temperature 'C' and four set to elevate night temperature 'H'. External controls comprised vines in the absence of closed-top chambers and fan heaters. Bunches were tagged at 50% capfall and treatments were assembled at three berry phenological stages: pre-véraison (E-L 31), véraison (E-L 34) and ripening (E-L 36), for eleven days each.

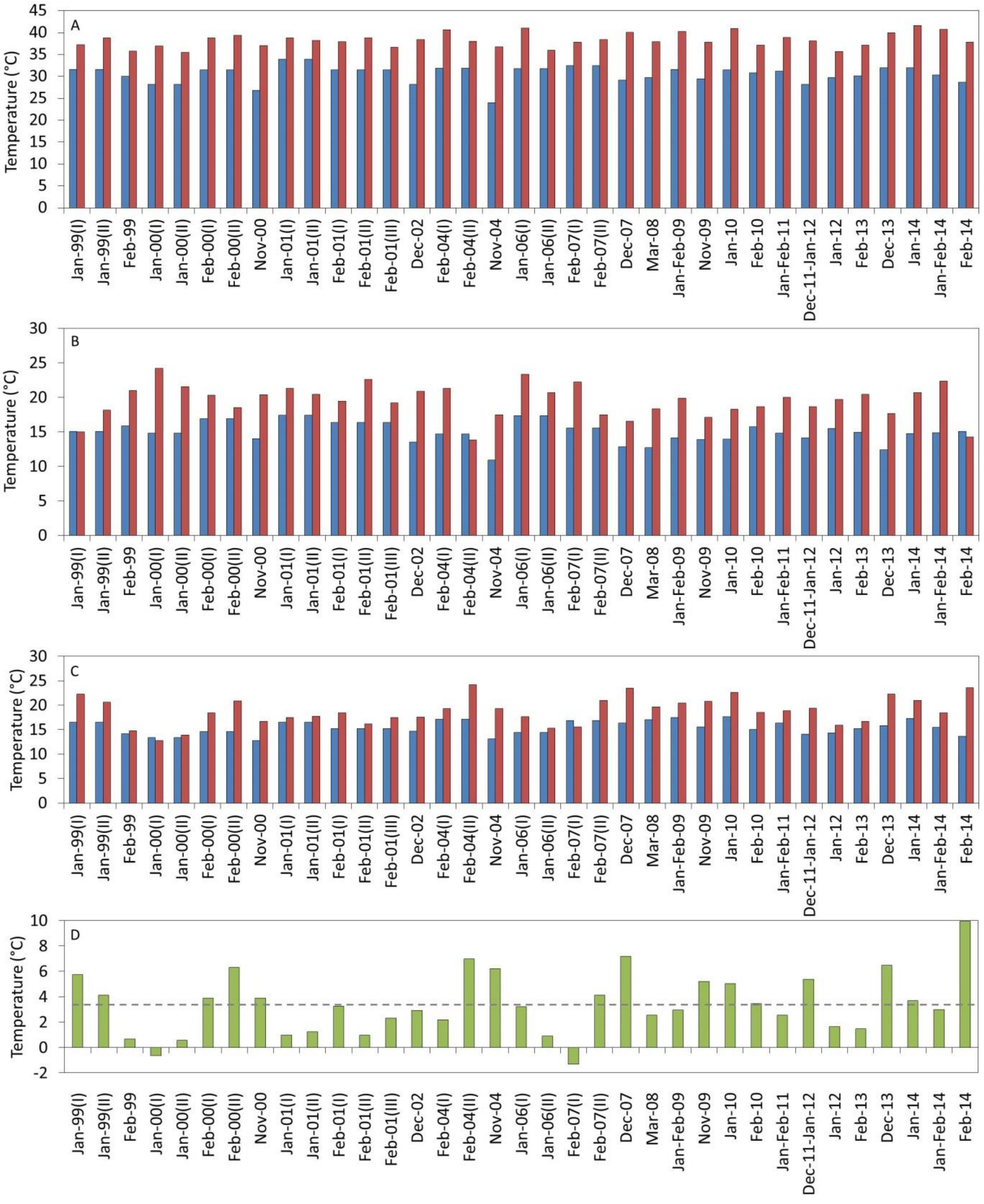

Figure S2: Temperatures during natural heatwaves and the monthly averages during South Australian summers from 1999-2014. Average (blue) and heatwave (red) temperatures for (A) maximum day, (B) minimum night and (C) diurnal range. (D) Difference between heatwave and average diurnal temperature ranges (dashed line indicates average difference of 3.4°C for the 15 year timeperiod). Data were recorded at the Nuriootpa Meteorological Station (ID 023373) by the Australian Bureau of Meteorology (BOM; http://www.bom.gov.au/climate/data-services). Heatwaves were defined as three or more consecutive days with a maximum temperature ≥ 35°C.

Average heatwave temperatures were calculated across the duration of the heatwave. Average temperatures were calculated for the corresponding months in which heatwaves occurred. Diurnal ranges were calculated as the average differences between maximum day temperature and minimum temperature from the previous night.

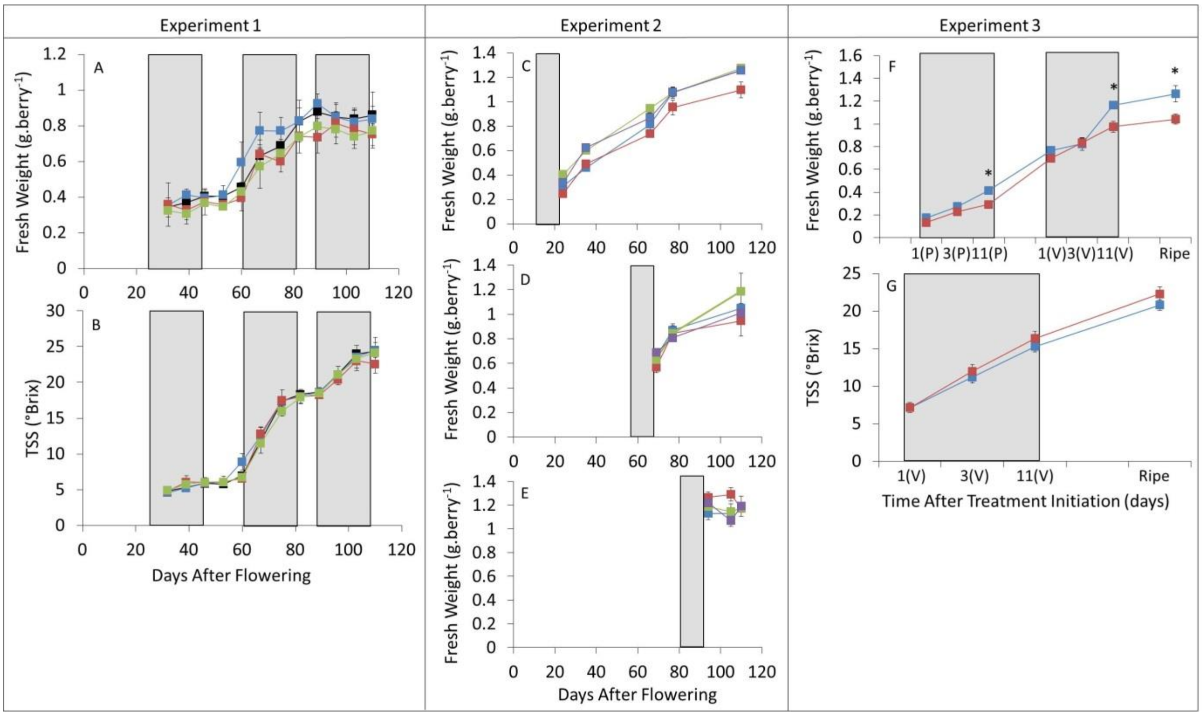

Figure S3: Effects of all elevated temperature treatments on berry fresh weight and TSS. (A) and (B) show berry fresh weight and TSS respectively, from control vines (black) and vines exposed to three-week warming treatments at pre-véraison (blue), véraison (red) and ripening (green) stages in the field (Experiment 1). (n=3 ± S.D.). (C), (D) and (E) show berry fresh weight for eleven-day heating treatments conducted at pre-véraison, véraison and ripening stages, respectively, in the field (Experiment 2), with vines exposed to control conditions (blue), or heating during the day-only (red), night-only (green), or both day and night (purple). (n=4 ± S.D.). (F) and (G) show berry fresh weight and TSS, respectively, for vines exposed to eleven-day control (blue) and heat (red) treatments in controlled-environment chambers (Experiment 3), plotted against the number of days after the initiation of pre-véraison [P] and véraison [V] treatments. (n=8 ± S.D.). *Significantly different from control (independent t-test, $p < 0.05$). Treatment periods for all experiments are highlighted in grey.

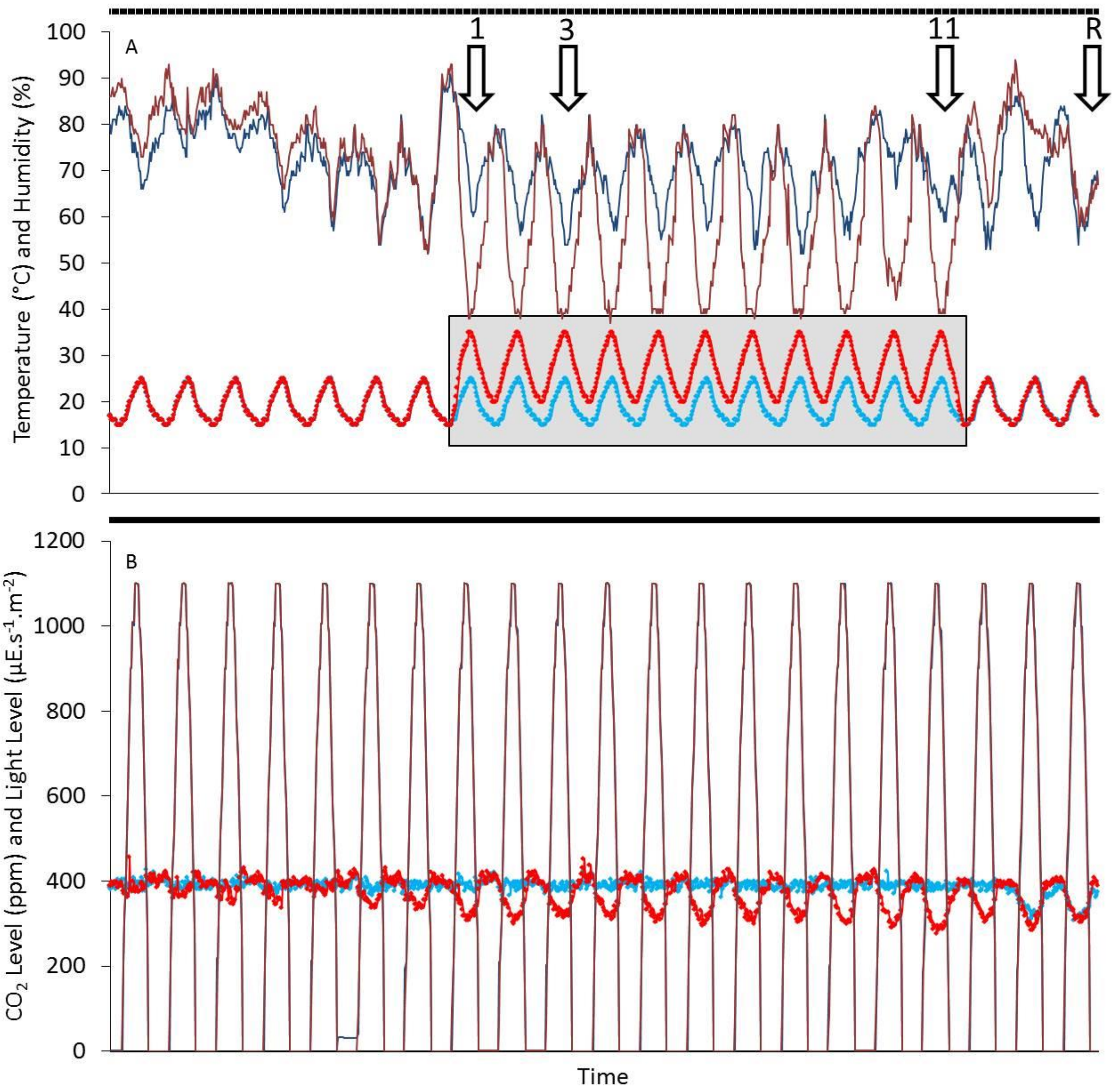

Figure S4: Controlled environment chamber growth conditions during véraison treatment for control and elevated temperature regimes (Experiment 3). Data from 30-minute intervals recorded within controlled-environment chambers containing potted Shiraz vines at The Plant Accelerator (The University of Adelaide, Waite campus). (A) Temperature (bright blue and red for control and heated chambers respectively) and humidity (dark blue and red for control and heated chambers respectively) and (B) CO_2 (bright blue and red) and light (dark blue and red) levels. Data were collected across the seven day equilibration period (both cabinets set to the control temperature regime, 25:15°C), eleven days of heating (one chamber set to elevated temperature regime, 35:20°C) and three days of recovery (both cabinets set to the control temperature regime). The heat treatment period is highlighted in grey. Arrows indicate sample collection points and the number of days from treatment initiation.

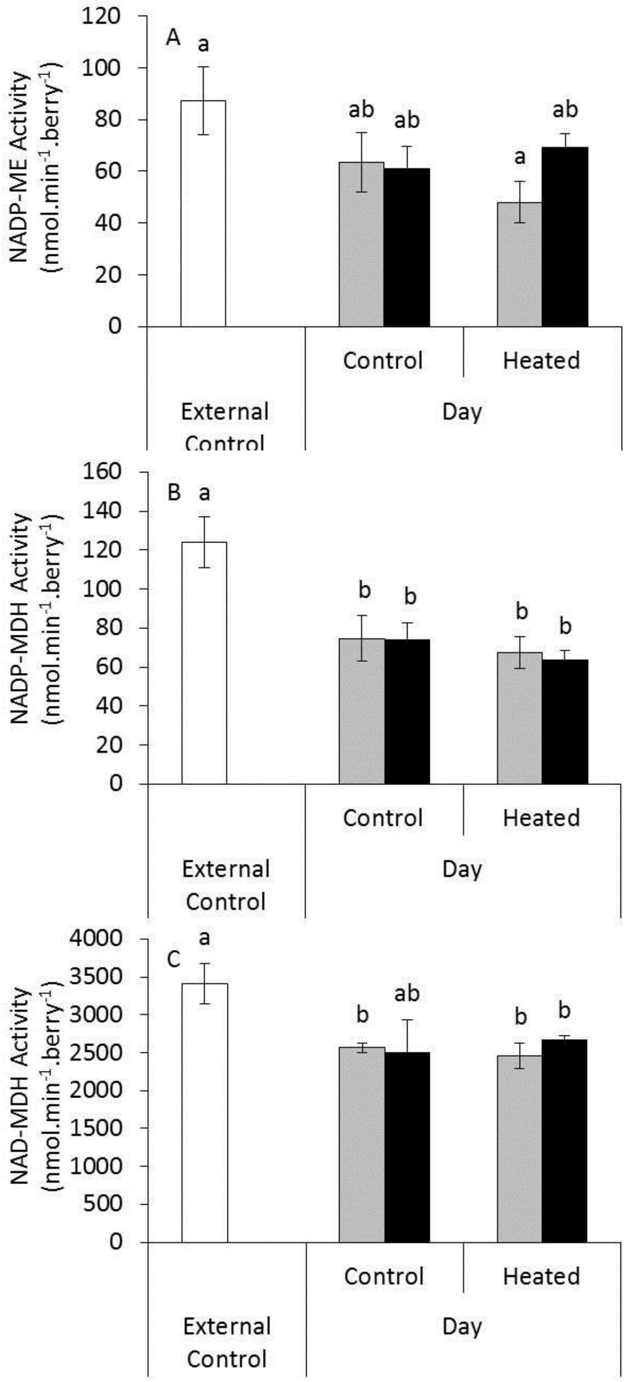

Figure S5: Activities of enzymes directly involved in malate metabolism following an eleven-day elevated temperature treatment at véraison (Experiment 2). Effects on (A) NADP-dependent malic enzyme activity, (B) NADP-dependent malate dehydrogenase activity and (C) NAD-dependent malate dehydrogenase activity. Control and heated day treatments are indicated on the x-axes, with different shades representing external controls (white), control nights (grey) and heated nights (black). (n=4 ± S.E.M.). Columns sharing a letter are not significantly different (two-way ANOVA with Tukey's multiple comparisons test; independent t-tests for comparison with external control, $p < 0.05$).
